# Editorial: Genetic, Epigenetic, and Epitranscriptomic Mechanisms Associated With Learning and Memory

**DOI:** 10.3389/fgene.2021.835719

**Published:** 2022-01-14

**Authors:** Anna R. Moore

**Affiliations:** Department of Biology, Temple University, Philadelphia, PA, United States

**Keywords:** genetics, epigenetics, epitranscriptome, learning and memory, plasticity

The formation of long-term memory requires activity-dependent gene expression and protein translation, which allow the neurons to dynamically adjust their synaptic strength during learning (Martin et al., 2000; Sutton and Schuman, 2006). One of the best-studied forms of synaptic plasticity is long-term potentiation (LTP), which produces a persistent increase in the synaptic strength of neurons (Martin et al., 2000). It is well established that messenger RNAs (mRNAs) and proteins must be synthesized to maintain the long-lasting increase in the magnitude of the synaptic response and memory consolidation (Hernandez and Abel, 2008; Yap and Greenberg, 2018). To date, large collections of genes have been associated with learning and memory processes (Knowles et al., 2014; Reshetnikov et al., 2020). Yet new gene transcription alone cannot justify the dynamic and long-term sustainability often observed with learning and memory. But how are these synaptic changes, which arise during learning, subsequently retained throughout life? For example, how does one remember to ride a bicycle after years of nonuse? How does our brain store and retain this information at the molecular level?

The traditional view of nature (i.e., genes) versus nurture (i.e., environment and experience) as two separate entities has been challenged over the past two decades by evidence of a direct and dynamic relationship between genes and environmental stimuli (Tsankova et al., 2007; Guan et al., 2015). Epigenetic regulation is the process by which the activity of a particular gene is controlled by the structure of nearby chromatin (Tsankova et al., 2007). This can occur via chromatin remodeling and involves histone modifications, DNA methylation and the binding of numerous transcription factors and transcriptional co-activators and co-repressors all of which are thought to play a critical role in retaining long-term changes in post-mitotic cells. Epigenetic regulation was originally highlighted to be crucial for nervous system development, but has also been identified in the mature brain and may underlie stables changes in gene expression both under normal conditions and in several neuropathological states. In the example of learning and memory, different epigenetic regulators work in concert to converge the upstream signaling cascade and manipulate downstream gene transcription with precise timing. In addition to epigenetic modifications, the emerging field of epitranscriptomics (RNA modifications) has also rapidly shifted our views on the mechanisms that regulate gene expression (Leighton et al., 2018; Widagdo et al., 2021). Following *de novo* gene expression, both mRNAs and non-coding RNAs (ncRNAs) (e.g., circular RNAs and micro RNAs) undergo further processing, including splicing, polyadenylation and nuclear export to ensure their proper expression, localization or translational control in neurons. Together the cross-talk between transcription factors and epigenetic modifiers play an important role in driving neuronal plasticity and long-term memory formation. However, the molecular mechanisms controlling both epigenetic and epitranscriptomic regulation remain largely unknown. In addition, molecular aberrations in these regulatory pathways are thought to contribute to various kinds of learning disability and memory deficits. This special topic covers a series of original research and review articles that further our knowledge about the genetic, epigenetic and epitranscriptomic mechanisms associated with learning and memory in health and disease.

Alzheimer’s disease (AD) is a neurodegenerative disorder which presents with memory deficits as well as complex genetic characteristics. Accumulating evidence suggests that both epigenetic and epitranscriptomics may play important roles in cognitive disorders like AD, yet how they contribute to the epidemiology of the disorder is not well understood. In an effort to gain better insight into the relationship between key AD-risk genes and cognitive severity Zhang et al., explored the impact of three major high-risk AD genes (*APOEε4*, *ABCA7*, and *CLU*) on function connectivity in healthy middle-aged adults. They found that both *CLU* and *ABCA7* might interact to regulate the functional connectivity of the medial prefrontal and posterior cingulate cortices and that *APOEε4* might also interact with *ABCA7* and *CLU* in middle-aged carriers to affect functional output and disease severity. While this paper focuses on DNA regulation, a complementary review paper by Wei et al., focuses on RNA regulation. This paper investigates the impairment of non-coding microRNAs (miRNAs, miRs) in AD pathogenesis and their potential to serve as biomarkers of disease progression. In addition, Wang et al., investigated the role of noncoding circular RNAs (cirRNAs), which have also been shown to play a role in learning and memory and neurological disorders such as AD and Down Syndrome. In this study, hippocampal fetal tissue from Down Syndrome patients was used to quantify circRNA-miRNA-mRNA competing endogenous RNA (ceRNA) regulatory networks. The authors identified several alterations in pathways associated with synaptic plasticity and learning and memory. This work suggests a potential role for noncoding RNAs, such as circRNAs, in neural signaling transfer might play an underlying role in disease progression.

Understanding how these circRNAs might be regulated in response to learning and memory paradigms can be gleamed from Dell’Orco et al. and colleagues. Here the investigators examine the interaction of the RNA binding protein (RBP) HuD (aka, ELAVL4) with circRNAs. After previously characterizing that HuD interacts with *circHomer1a*, the authors went on in the current work to demonstrate that HuD also binds to 226 other genes involved in neuronal differentiation, synapse formation and learning and memory. Together, HuD binds to and regulates the levels of multiple circRNAs to ensure changes in HuD-regulated ceRNA networks to modulate synaptic plasticity. Finally, regulation of mRNA splicing by RBPs can also affect synaptic plasticity and disease progression in the brain. In a review by Gallo et al. and collogues, Apolipoprotein E receptor 2 (apoER2), a type I transmembrane protein of the low-density lipoprotein receptor family known to regulate learning and memory in the adult brain regulation is discussed. ApoER2 has several known isoforms whose splice variants are cell type specific. A deeper understanding on the effects of RBPs on alternative splicing and the epigenetic factors modulating RBPs themselves is still needed to further understand the contribution of specific regulatory pathways. In the future it will be critical to link different DNA and RNA regulatory pathways to both learning and memory paradigms and disease pathogenesis. In order to do so, a thorough understanding is needed of how these different signaling components work together with environmental influences to shape brain plasticity and store long-term memories.
